# Supplementary material for: Aspergillus fumigatus Supernatants Disrupt Bronchial Epithelial Monolayers: Potential Role for Enhanced Invasion in Cystic Fibrosis
Source: J Fungi (Basel). 2023 Apr 19;9(4):490. doi: 10.3390/jof9040490 (PMC10141846; doi:10.3390/jof9040490)
Supplement: Supplementary file 1 [file jof-09-00490-s001.zip › jof-2283803-supplementary.pdf]

STRAf

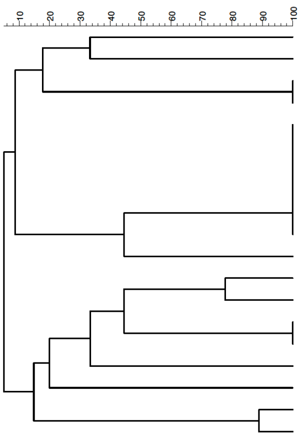

STRAf

2A	2B	2C	3A	3B	3C	4A	4B	4C	
18.0	16.0	24.0	35.0	11.0	20.0	10.0	9.0	8.0	47
19.0	21.0	11.0	22.0	11.0	23.0	10.0	8.0	8.0	50(m10)
25.0	18.0	11.0	30.0	23.0	7.0		11.0	8.0	45(m0_1)
25.0	18.0	11.0	30.0	23.0	7.0	13.3	11.0	8.0	45(m0_2)
17.0	24.0	10.0	40.0	11.0	29.0	25.0	9.0	5.0	49
17.0	24.0	10.0	40.0	11.0	29.0	25.0	9.0	5.0	50(m2)
17.0	24.0	10.0	40.0	11.0	29.0	25.0	9.0	5.0	54(m0)
17.0	24.0	10.0	40.0	11.0	29.0	25.0	9.0	5.0	54(m2)
17.0	24.0	10.0	40.0	11.0	29.0	25.0	9.0	5.0	54(m4_1)
17.0	24.0	10.0	40.0	11.0	29.0	25.0	9.0	5.0	54(m4_2) AF1
17.0	24.0	10.0	12.0	11.0	22.0	10.0	13.0	10.0	51(m4)
26.0	20.0	15.0	34.0	9.0	7.0	9.0	10.0	10.0	48
14.0	20.0	8.0	34.0	9.0	7.0	9.0	10.0	10.0	51(m2) AF2
14.0	20.0	8.0	28.0	9.0	8.0	8.0	10.0	26.0	52
14.0	20.0	8.0	28.0	9.0	8.0	8.0	10.0	26.0	53
14.0	20.0	8.0	27.0	10.0	21.0	8.0	8.0	5.0	51(m0)
26.0	20.0	15.0	48.0	19.0	7.0	13.3	9.0	7.0	50(m4)
18.0	12.0	8.0	39.0	10.0	27.0	9.0	9.0	9.0	46(m2)
18.0	12.0	8.0	39.0	10.0	26.2	9.0	9.0	9.0	46(m4)

Figure S1. STRAf assay typing of A. fumigatus isolates from CF patients. Numbers represent patients and m stands for month of sample taken. Marked isolates were used in this study. Patient 54 had samples taken at months 0, 2 and 4 (two colonies picked) and had indistinguishable A. fumigatus isolates at all sampling times. Isolate 54(m4_2) was used as the persistent isolate (AF1) in this study. Patient 51 had samples taken at months 0, 2 and 4 months and had distinguishable isolates at each sampling time. Isolate 51(m2) was used as the non-persistent isolate (AF2) in this study.

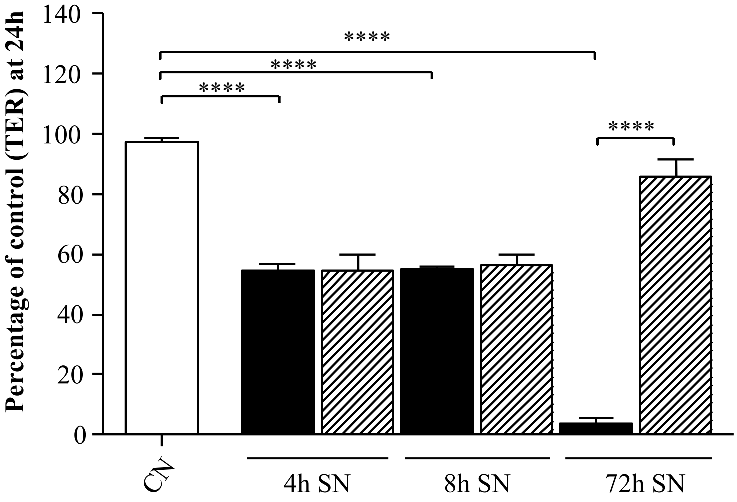

Figure S2. Effect of supernatants from A. fumigatus and ΔgliG cultures on HBE monolayer integrity. TEERs of HBE monolayers following 24 h exposure to supernatants of 4, 8 and 72 h cultures of Af293 (black bars) and ΔgliG (bars with diagonal lines). The TEER values of untreated HBE monolayers (CN, white bar) at 24 h were included. Error bars represent standard error of at least three independent replicates. **** $p < 0.0001$.
